# 1,3-Benzothia­zole–oxalic acid (2/1)

**DOI:** 10.1107/S1600536811032260

**Published:** 2011-08-17

**Authors:** Ashraf Ahmad Ali Abdalsalam, Mohammad T.M. Al-Dajani, Nornisah Mohamed, Madhukar Hemamalini, Hoong-Kun Fun

**Affiliations:** a School of Pharmaceutical Sciences, Universiti Sains Malaysia, 11800 USM, Penang, Malaysia; b X-ray Crystallography Unit, School of Physics, Universiti Sains Malaysia, 11800 USM, Penang, Malaysia

## Abstract

The asymmetric unit of the title compound, C_7_H_5_NS·0.5C_2_H_2_O_4_, contains one benzothia­zole mol­ecule and half an oxalic acid mol­ecule, the complete mol­ecule being generated by inversion symmetry. The benzothia­zole mol­ecule is essentially planar, with a maximum deviation of 0.007 (1) Å. In the crystal, the benzothia­zole mol­ecules inter­act with the oxalic acid mol­ecules *via* O—H⋯N and C—H⋯O hydrogen bonds generating *R*_2_^2^(8) (× 2) and *R*_4_^4^(10) motifs, thereby forming supra­molecular ribbons along [101].

## Related literature

For background to the biological activity of benzothia­zoles, see: Bradshaw *et al.* (1998 ▶); Dögruer *et al.* (1998 ▶); Dash *et al.* (1980 ▶); Cox *et al.* (1982 ▶).
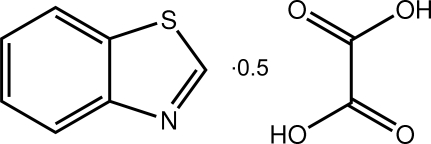


## Experimental

### 

#### Crystal data

C_7_H_5_NS·0.5C_2_H_2_O_4_
*M* *_r_* = 180.20
Monoclinic, 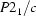

*a* = 4.0231 (3) Å
*b* = 26.039 (2) Å
*c* = 8.5605 (6) Å
β = 116.064 (3)°
*V* = 805.58 (10) Å^3^
*Z* = 4
Mo *K*α radiation
μ = 0.35 mm^−1^
*T* = 296 K
0.62 × 0.40 × 0.04 mm

#### Data collection

Bruker APEXII DUO CCD area-detector diffractometer
Absorption correction: multi-scan (*SADABS*; Bruker, 2009 ▶) *T*_min_ = 0.811, *T*_max_ = 0.985
10970 measured reflections
3204 independent reflections
2417 reflections with *I* > 2σ(*I*)
*R*_int_ = 0.026

#### Refinement

*R*[*F* ^2^ > 2σ(*F* ^2^)] = 0.038
*wR*(*F* ^2^) = 0.114
*S* = 1.04
3204 reflections
112 parameters
H atoms treated by a mixture of independent and constrained refinement
Δρ_max_ = 0.36 e Å^−3^
Δρ_min_ = −0.23 e Å^−3^

### 

Data collection: *APEX2* (Bruker, 2009 ▶); cell refinement: *SAINT* (Bruker, 2009 ▶); data reduction: *SAINT*; program(s) used to solve structure: *SHELXTL* (Sheldrick, 2008 ▶); program(s) used to refine structure: *SHELXTL*; molecular graphics: *SHELXTL*; software used to prepare material for publication: *SHELXTL* and *PLATON* (Spek, 2009 ▶).

## Supplementary Material

Crystal structure: contains datablock(s) global, I. DOI: 10.1107/S1600536811032260/tk2778sup1.cif

Structure factors: contains datablock(s) I. DOI: 10.1107/S1600536811032260/tk2778Isup2.hkl

Supplementary material file. DOI: 10.1107/S1600536811032260/tk2778Isup3.cml

Additional supplementary materials:  crystallographic information; 3D view; checkCIF report

## Figures and Tables

**Table 1:** Hydrogen-bond geometry (Å, °)

*D*—H⋯*A*	*D*—H	H⋯*A*	*D*⋯*A*	*D*—H⋯*A*
O2—H1*O*2⋯N1	0.89 (2)	1.80 (2)	2.6663 (15)	166 (2)
C5—H5*A*⋯O1	0.93	2.48	3.3263 (17)	151
C7—H7*A*⋯O2^i^	0.93	2.48	3.4029 (18)	170

Symmetry code: (i) 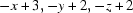.

**Fig. 1.**
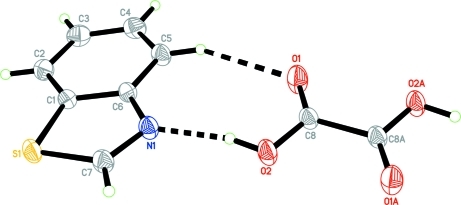
Contents of (I) showing the molecule of benzothiazole and the full molecule of oxalic acid after the application of inversion symmetry (A: -x+2, -y+2, -z+1). The atoms are displayed with 50% probability displacement ellipsoids and the atom-numbering scheme. Hydrogen bonds are shown as dashed lines.

**Fig. 2.**
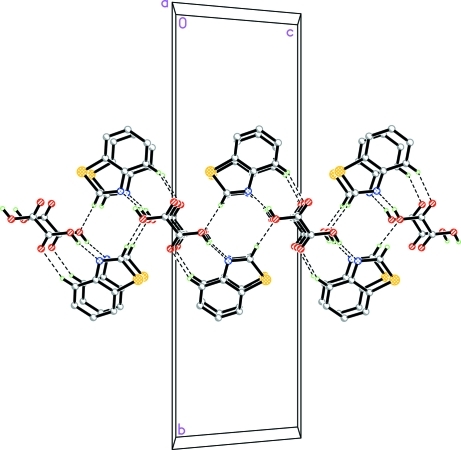
Partial crystal packing in (I) with dashed lines representing hydrogen bonding.

## supplementary crystallographic information

### Comment

Benzothiazoles are used as anti-neoplastic agents and show anti-nociceptive, anti-inflammatory and anti-tumour activities (Bradshaw *et al.*, 1998; Dögruer *et al.*, 1998). Some Schiff bases derived from thiazole and benzothiazoles (Dash *et al.*, 1980) and several derivatives of the styryl-benzothiazoles have also shown biological activity (Cox *et al.*, 1982). In view of the above biological activities associated with the benzothiazole, herein, we present the title compound (I), extracted from the juice of Guava (*Psidium guajava*).

The asymmetric unit of the title compound, (I), contains one benzothiazole molecule and a half of an oxalic acid molecule (which lies on an inversion centre) as detailed in Fig. 1. The benzothiazole (N1/S1/C1–C7) molecule is essentially planar, with a maximum deviation of 0.007 (1) Å for atom C6.

In the crystal structure, Fig. 2, the benzothiazole molecules interact with the oxalic acid molecules *via* O—H···N and C—H···O hydrogen bonds (Table 1) generating *R*^2^_2_(8) and *R*^4^_4_(10) motifs and forming supramolecular ribbons along the [1 0 1] direction.

### Experimental

The juice of Guava (*Psidium guajava*) was extracted using soxhlet extraction method with methanol as solvent. After 24 hours at room temperature, a precipitate was formed and the filtrate removed. The precipitate was washed by using a mixture (90–100) ml of n-hexane-ethyl acetate. It was recrystallized by dissolving in methanol. Brown crystals were formed which melted at *M*.pt 323 K.

### Refinement

Atom H1O2 was located from a difference Fourier map and refined with *U*_iso_(H) = 1.5*U*_eq_(O) [O—H = 0.89 (2) Å]. The remaining H atoms were positioned geometrically [C—H = 0.93 Å] and were refined using a riding model, with *U*_iso_(H) = 1.2*U*_eq_(C).

### Crystal data

**Table:**

C_7_H_5_NS·0.5C_2_H_2_O_4_	*F*(000) = 372
*M**_r_* = 180.20	*D*_x_ = 1.486 Mg m^−^^3^
Monoclinic, *P*2_1_/*c*	Mo *K*α radiation, λ = 0.71073 Å
Hall symbol: -P 2ybc	Cell parameters from 3803 reflections
*a* = 4.0231 (3) Å	θ = 2.8–32.6°
*b* = 26.039 (2) Å	µ = 0.35 mm^−^^1^
*c* = 8.5605 (6) Å	*T* = 296 K
β = 116.064 (3)°	Plate, brown
*V* = 805.58 (10) Å^3^	0.62 × 0.40 × 0.04 mm
*Z* = 4	

### Data collection

**Table:**

Bruker APEXII DUO CCD area-detector diffractometer	3204 independent reflections
Radiation source: fine-focus sealed tube	2417 reflections with *I* > 2σ(*I*)
graphite	*R*_int_ = 0.026
φ and ω scans	θ_max_ = 33.9°, θ_min_ = 2.8°
Absorption correction: multi-scan (*SADABS*; Bruker, 2009)	*h* = −6→6
*T*_min_ = 0.811, *T*_max_ = 0.985	*k* = −40→40
10970 measured reflections	*l* = −13→13

### Refinement

**Table:**

Refinement on *F*^2^	Primary atom site location: structure-invariant direct methods
Least-squares matrix: full	Secondary atom site location: difference Fourier map
*R*[*F*^2^ > 2σ(*F*^2^)] = 0.038	Hydrogen site location: inferred from neighbouring sites
*wR*(*F*^2^) = 0.114	H atoms treated by a mixture of independent and constrained refinement
*S* = 1.04	*w* = 1/[σ^2^(*F*_o_^2^) + (0.0557*P*)^2^ + 0.1124*P*] where *P* = (*F*_o_^2^ + 2*F*_c_^2^)/3
3204 reflections	(Δ/σ)_max_ = 0.001
112 parameters	Δρ_max_ = 0.36 e Å^−^^3^
0 restraints	Δρ_min_ = −0.23 e Å^−^^3^

### Special details

**Table:**

Geometry. All s.u.'s (except the s.u. in the dihedral angle between two l.s. planes) are estimated using the full covariance matrix. The cell s.u.'s are taken into account individually in the estimation of s.u.'s in distances, angles and torsion angles; correlations between s.u.'s in cell parameters are only used when they are defined by crystal symmetry. An approximate (isotropic) treatment of cell s.u.'s is used for estimating s.u.'s involving l.s. planes.
Refinement. Refinement of F^2^ against ALL reflections. The weighted R-factor wR and goodness of fit S are based on F^2^, conventional R-factors R are based on F, with F set to zero for negative F^2^. The threshold expression of F^2^ > 2σ(F^2^) is used only for calculating R-factors(gt) etc. and is not relevant to the choice of reflections for refinement. R-factors based on F^2^ are statistically about twice as large as those based on F, and R- factors based on ALL data will be even larger.

### Fractional atomic coordinates and isotropic or equivalent isotropic displacement parameters (Å^2^)

**Table:**

	*x*	*y*	*z*	*U*_iso_*/*U*_eq_	
S1	1.14317 (9)	0.875823 (14)	1.21487 (4)	0.04461 (11)	
N1	1.0034 (3)	0.91965 (4)	0.92292 (13)	0.0385 (2)	
C1	0.8353 (3)	0.84567 (4)	1.02665 (14)	0.0350 (2)	
C2	0.6466 (4)	0.79950 (5)	1.00741 (19)	0.0448 (3)	
H2A	0.6763	0.7800	1.1037	0.054*	
C3	0.4144 (4)	0.78360 (5)	0.8411 (2)	0.0493 (3)	
H3A	0.2881	0.7527	0.8252	0.059*	
C4	0.3652 (4)	0.81302 (5)	0.69656 (18)	0.0468 (3)	
H4A	0.2035	0.8017	0.5861	0.056*	
C5	0.5516 (4)	0.85854 (5)	0.71444 (15)	0.0412 (3)	
H5A	0.5187	0.8780	0.6176	0.049*	
C6	0.7919 (3)	0.87487 (4)	0.88198 (14)	0.0329 (2)	
C7	1.1947 (4)	0.92418 (5)	1.09000 (16)	0.0408 (3)	
H7A	1.3510	0.9520	1.1395	0.049*	
O1	0.7128 (3)	0.94803 (4)	0.47893 (12)	0.0584 (3)	
O2	1.1410 (3)	0.98494 (4)	0.71853 (11)	0.0468 (2)	
H1O2	1.064 (6)	0.9617 (8)	0.771 (3)	0.070*	
C8	0.9467 (3)	0.97976 (4)	0.55062 (14)	0.0363 (2)	

### Atomic displacement parameters (Å^2^)

**Table:**

	*U*^11^	*U*^22^	*U*^33^	*U*^12^	*U*^13^	*U*^23^
S1	0.04724 (18)	0.0555 (2)	0.02518 (14)	−0.00784 (13)	0.01050 (12)	0.00106 (11)
N1	0.0444 (5)	0.0389 (5)	0.0286 (4)	−0.0058 (4)	0.0128 (4)	−0.0010 (3)
C1	0.0333 (5)	0.0404 (5)	0.0298 (5)	0.0001 (4)	0.0123 (4)	0.0004 (4)
C2	0.0428 (6)	0.0456 (6)	0.0446 (6)	−0.0034 (5)	0.0179 (5)	0.0069 (5)
C3	0.0464 (7)	0.0420 (6)	0.0565 (8)	−0.0098 (5)	0.0199 (6)	−0.0064 (6)
C4	0.0442 (6)	0.0515 (7)	0.0390 (6)	−0.0080 (5)	0.0131 (5)	−0.0136 (5)
C5	0.0447 (6)	0.0465 (6)	0.0281 (5)	−0.0032 (5)	0.0121 (5)	−0.0042 (4)
C6	0.0349 (5)	0.0351 (5)	0.0275 (5)	−0.0006 (4)	0.0126 (4)	−0.0019 (4)
C7	0.0442 (6)	0.0426 (6)	0.0315 (5)	−0.0082 (5)	0.0129 (5)	−0.0038 (4)
O1	0.0705 (7)	0.0600 (6)	0.0318 (4)	−0.0320 (5)	0.0108 (4)	−0.0010 (4)
O2	0.0557 (5)	0.0486 (5)	0.0255 (4)	−0.0169 (4)	0.0081 (4)	0.0029 (3)
C8	0.0400 (5)	0.0365 (5)	0.0265 (5)	−0.0049 (4)	0.0092 (4)	0.0009 (4)

### Geometric parameters (Å, °)

**Table:**

S1—C7	1.7222 (13)	C4—C5	1.3748 (19)
S1—C1	1.7300 (12)	C4—H4A	0.9300
N1—C7	1.2979 (15)	C5—C6	1.3979 (15)
N1—C6	1.3944 (14)	C5—H5A	0.9300
C1—C2	1.3926 (17)	C7—H7A	0.9300
C1—C6	1.3972 (15)	O1—C8	1.1989 (14)
C2—C3	1.379 (2)	O2—C8	1.3068 (13)
C2—H2A	0.9300	O2—H1O2	0.89 (2)
C3—C4	1.394 (2)	C8—C8^i^	1.540 (2)
C3—H3A	0.9300		
			
C7—S1—C1	89.19 (6)	C4—C5—C6	118.30 (12)
C7—N1—C6	110.72 (10)	C4—C5—H5A	120.9
C2—C1—C6	121.04 (11)	C6—C5—H5A	120.9
C2—C1—S1	129.19 (10)	N1—C6—C1	114.05 (10)
C6—C1—S1	109.76 (8)	N1—C6—C5	125.65 (10)
C3—C2—C1	117.92 (12)	C1—C6—C5	120.30 (11)
C3—C2—H2A	121.0	N1—C7—S1	116.28 (9)
C1—C2—H2A	121.0	N1—C7—H7A	121.9
C2—C3—C4	121.26 (12)	S1—C7—H7A	121.9
C2—C3—H3A	119.4	C8—O2—H1O2	108.3 (15)
C4—C3—H3A	119.4	O1—C8—O2	126.13 (11)
C5—C4—C3	121.16 (12)	O1—C8—C8^i^	122.24 (12)
C5—C4—H4A	119.4	O2—C8—C8^i^	111.63 (12)
C3—C4—H4A	119.4		
			
C7—S1—C1—C2	179.27 (13)	C2—C1—C6—N1	−179.17 (11)
C7—S1—C1—C6	−0.42 (9)	S1—C1—C6—N1	0.55 (13)
C6—C1—C2—C3	−0.3 (2)	C2—C1—C6—C5	1.12 (18)
S1—C1—C2—C3	−179.91 (11)	S1—C1—C6—C5	−179.16 (9)
C1—C2—C3—C4	−0.9 (2)	C4—C5—C6—N1	179.48 (12)
C2—C3—C4—C5	1.1 (2)	C4—C5—C6—C1	−0.85 (19)
C3—C4—C5—C6	−0.3 (2)	C6—N1—C7—S1	0.05 (15)
C7—N1—C6—C1	−0.39 (15)	C1—S1—C7—N1	0.22 (11)
C7—N1—C6—C5	179.30 (12)		

Symmetry codes: (i) −*x*+2, −*y*+2, −*z*+1.

### Hydrogen-bond geometry (Å, °)

**Table:**

*D*—H···*A*	*D*—H	H···*A*	*D*···*A*	*D*—H···*A*
O2—H1O2···N1	0.89 (2)	1.80 (2)	2.6663 (15)	166 (2)
C5—H5A···O1	0.93	2.48	3.3263 (17)	151
C7—H7A···O2^ii^	0.93	2.48	3.4029 (18)	170

Symmetry codes: (ii) −*x*+3, −*y*+2, −*z*+2.
